# COVID-19 Pandemic: A Neurological Perspective

**DOI:** 10.7759/cureus.7889

**Published:** 2020-04-29

**Authors:** Durjoy Lahiri, Alfredo Ardila

**Affiliations:** 1 Neurology, Institute of Post Graduate Medical Education & Research, Kolkata, IND; 2 Neuropsychology, I.M. Sechenov First Moscow State Medical University, Moscow, RUS

**Keywords:** covid-19, neurological manifestations, viral infection

## Abstract

Even though severe acute respiratory syndrome coronavirus 2 (SARS-CoV-2) has been observed to principally affect the respiratory system, neurological involvements have already been reported in some published work. We have reviewed original articles, case reports, and existing open-source data-sets to delineate the spectrum of neurological disorders potentially observed in SARS-CoV-2 positive cases. Neurological involvement in coronavirus disease 2019 (COVID-19) corresponds to three situations: (a) neurological manifestations of viral infection, (b) post-infective neurological complications, and (c) infection in patients with neurological co-morbidity. Neurological manifestations can further be subdivided into the central nervous system (headache, dizziness, alteration of the sensorium, ataxia encephalitis, stroke, and seizures) and peripheral nervous system (skeletal muscle injury and peripheral nerve involvement including hyposmia and hypogeusia) symptomatology. Post-infective neurological complications include demyelinating conditions. Reduced mobility and dementia as co-morbidities may predispose a patient to have a viral infection. It is concluded that the pandemic of COVID-19 presents for a neurologist some unique challenges. We observe that SARS-CoV-2 may have various neurological manifestations and in many cases, neurological features may precede typical respiratory symptoms.

## Introduction and background

Coronavirus disease 2019 (COVID-19) has been declared a pandemic on the 11th of March, 2020 by the World Health Organization. The epicenter of this pandemic has shifted in quick succession from China to Europe to the United States of America in a matter of weeks. Since the middle of March 2020, South-east Asia has also seen a rise in the number of affected persons and it remains to be seen if there will be another twist in the story of this pandemic. One of the essential weapons to fight a pandemic of this stature is to gather as much knowledge as possible about the transmission dynamics and clinical manifestations while the quest for an effective vaccine keeps continuing.

Even though severe acute respiratory syndrome coronavirus 2 (SARS-CoV-2) has been observed to mainly affect the respiratory system, neurological involvements have already been reported in some published work. Several medical news bulletins, blogs, and articles across the globe have also raised concerns about brain invasion by this particular strain of coronavirus. This is actually not surprising given our previous experience of neuro-invasion by severe acute respiratory syndrome coronavirus (SARS-CoV) and the Middle East respiratory syndrome-related coronavirus (MERS-CoV). The body of literature on neurological aspects of SARS-CoV-2 is small but growing. However, we believe, an organized summary of the available information at this point would be indispensable to neurologists across the globe. Being well informed about the neurological presentations would not only help them have a high index of clinical suspicion but also take necessary precautions. Therefore, a brief literature review along with a critical appraisal of the evidence gathered so far is presented here from the perspective of a neurologist.

In the present paper, we have reviewed the recently published or pre-print original articles, case reports, and existing open-source data-sets in order to delineate the spectrum of neurological disorders in SARS-CoV-2 positive cases.

## Review

Mechanism of neuro-invasion

The putative mechanisms described to explain the neuro-invasion by a coronavirus (and one similar another RNA virus, influenza A) are hematogenous spread and retrograde axonal transport [1-4]. However, in light of contemporary evidence, some of the other possible routes of neuroinvasion by the SARS-CoV-2 deserve to be mentioned. Direct viral invasion of the brain leading to clinical encephalitis has been suspected after the treatment team of Beijing Ditan Hospital confirmed the presence of SARS-CoV-2 in the cerebrospinal fluid (CSF) of patients with COVID-19 by genome sequencing [5]. COVID-19 is widely known to cause respiratory insufficiency, and therefore, hypoxia needs to be considered among the major putative mechanisms of brain injury [6]. Cytokine storm, which is a well known immune reaction of this particular viral infection, may lead to inflammation and injury of the central nervous system (CNS) tissue. This idea is further supported by the observation that interleukin (IL)-6, an important member of the cytokine storm, is positively correlated with the severity of COVID-19 symptoms [7]. The affinity of the viral particle towards angiotensin-converting enzyme-2 (ACE-2), a cardio-cerebral vascular protection factor, has been in discussion in recent papers [8]. The expression of ACE-2 in the nervous system and skeletal muscles can indeed explain some of the neurological features reported so far. It has been postulated that the viral attachment to the ACE-2 at the level of the blood-brain barrier may jeopardize the protective mechanism surrounding the encephalon, giving way to viral encephalitis. In a similar vein, spinal cord membranes expressing ACE-2 can culminate into myelitis-like features following SARS-CoV-2 infection. Concern has also been raised that the viral particles binding to ACE-2 in cerebral blood vessels may actually raise the luminal pressure of those vessels leading to intracerebral hemorrhage [9]. Thus there are multiple mechanisms elaborated so far in the available literature that can explain the observed as well as anticipated neurological features of this ailment.

Classification and features of neurological involvement

Early data on COVID-19 suggest neurological involvement in a variable percentage of cases with particular expression in more severe patients [10]. Neurological involvement in COVID-19 can be discussed in three sections: (1) neurological features of viral infection, (2) post-infective neurological complications, (3) infection in patients with neurological co-morbidity. Another issue that deserves to be mentioned is the precautions to be taken for neurologically ill patients who require immunosuppressive agents. The latter group mostly consists of multiple sclerosis, myasthenia gravis, and autoimmune encephalitis.

Neurological features of viral invasion

Neurological manifestations of viral infection reported so far can further be subdivided into CNS and peripheral nervous system (PNS) features. CNS features include headache, dizziness, ataxia, alteration of sensorium, encephalitis, stroke, and seizures, while PNS features mostly refer to skeletal muscle injury and peripheral nerve involvement in the form of hyposmia and hypogeusia.

Headache can be a symptom of viral infection and usually remains associated with fever. Studies have reported an incidence of headaches ranging from 6 to 13% in COVID-19 cases [10-13]. However, concerns have already been raised in recent correspondence if this particular symptom is a manifestation of viral meningitis or, for that matter, encephalitis, which may reveal itself subsequently in the form of drowsiness and seizures. Japanese colleagues shared their recent experience in dealing with a young male patient without any contact or travel history who presented features of meningitis before being diagnosed with the infection of SARS-CoV-2 [14]. The issue becomes more substantial as one considers the report of virus detection in CSF of a COVID-19 patient. Another very recent report describes the occurrence of acute hemorrhagic necrotizing encephalopathy in COVID-19 patient [15]. In this particular case, however, the CSF examination for SARS-CoV-2 was not carried out, although bacterial culture and tests for other viruses were essentially negative. Therefore, neurologist needs to pay attention and analyze even a simple symptom such as headache in a known COVID-19 case, especially if the headache is out of proportion to rise in temperature and is associated with certain other features such as vomiting and altered sensorium.

Another noteworthy observation is the occurrence of cerebrovascular events (CVE) associated with this ailment. The initial retrospective case series study from Wuhan, China, reported that 5.7% (5/76) of the cases showing neurological involvement could be attributed to acute CVE [10]. Notably, four cases had an ischemic stroke, while cerebral hemorrhage was found in a single patient who died later on. Another recent paper from the same center, which analyzed 221 participants, elaborates that 5.88% (13/221) cases had some sort of new-onset CVE [16]. The majority of them presented acute ischemic stroke (11 patients), while hemorrhagic stroke (one patient) and cerebral venous sinus thrombosis (one patient) were also found but uncommonly. Therefore, thrombotic manifestations were way more frequent than hemorrhage. This observation may possibly be linked to the finding that patients with CVE were more likely to have enhanced inflammatory response as reflected in their C-reactive protein (CRP) and D-dimer levels. It is within the realm of possibilities that the viral infection may have given way to an inflammatory storm that ultimately culminated in accelerated thrombosis. The term "accelerated thrombosis" seems more meaningful if seen in the context of another finding in this particular retrospective study. Patients with CVE were not only significantly older than those without CVE but also were more likely to have pre-existent vascular risk factors. This observation is particularly relevant in the Indian scenario because vascular risk factors are common in the population, and so is the incidence of stroke. Moreover, any viral infection in a stroke patient not only delays recovery but also may actually worsen the neurological deficit, the latter being sometimes attributed to hemorrhagic transformation. The mortality rate was indeed found to be higher in COVID-19 stroke patients, with 38% indicating a worse prognosis in this group of patients. The above discussion brings out the two-way relationship between COVID-19 and stroke, which a neurologist has to bear in mind while attending patients in these difficult circumstances. The issue becomes more complicated when one considers treating a patient with ischemic stroke in the background of coronavirus infection. The application of antiplatelets and anticoagulants is tricky because the virus is known to cause prominent respiratory involvement, and according to some authors, the involvement of the nervous system may be partly responsible for respiratory impairment.

Impaired consciousness has so far been reported in 7.5% hospitalized patients of COVID-19 [10]. Severely affected patients are more likely to present impaired consciousness. There can be multiple underlying reasons for a patient with COVID-19 to present with altered sensorium, which include viral encephalitis, metabolic perturbation, infectious toxic encephalopathy, seizures with post-ictal confusion, and stroke (either strategic area involvement or large lesion with edema). A recent document, (published in Neurology Today Online) by Italian neurologists (addressed to their US colleagues) mentions the importance of recognizing poorly defined neurological features in confirmed as well as presumptive cases of COVID-19. Among these symptoms, altered sensorium indeed has drawn the attention of the neurologists mostly because it delays diagnostic procedures as the virus is widely known to be a respiratory pathogen. A detailed look at the description of a single case of encephalopathy in association with COVID-19 reveals that an altered level of consciousness may precede typical respiratory symptoms by days [17]. Given the transmission dynamics of the infection, this information is extremely important as health care providers at the emergency department may get inadvertent exposure while handling patients with altered sensorium. In a consecutive series of COVID-19 related acute respiratory distress syndrome (ARDS) patients (n=58) reported from France, altered consciousness, including agitation and confusion, has been documented in more than two-thirds of the cases. Additionally, 67% of the recruited patients in this study had prominent cortico-spinal signs [18]. In this context, telephonic conversation with the physician in charge of an infectious disease hospital in Kolkata (India) reveals that headache is more of a generalized symptom in both old and young patients, while delirium is a frequent presentation among the elderly. The hospital presently is catering to more than 60 confirmed cases of COVID-19, while around 250 presumptive cases are in isolation.

In the Wuhan study, ataxia was found in only one patient, although a detailed description or anatomical substrate of this particular symptom was not available in this paper [10]. We at our center recently encountered a 72-year-old man presenting with acute onset cerebellar ataxia followed by encephalopathy, who was finally diagnosed with SARS-CoV-2 induced pneumonia. Notably, in our patient neurological manifestation preceded respiratory features by days.

The association of seizure and COVID-19 can be multi-faceted. An early report suggested a very low incidence (0.5%) of seizure disorder [10]. However, as numbers start growing across the globe, this issue is anticipated to become a non-negligible one. Firstly, a seizure may be a manifestation of viral invasion into the CNS. Secondly, this particular infection is known to cause fatal pneumonia that gives way to severe hypoxemia, which may result in brain injury and seizures thereof. Metabolic perturbations and septic encephalopathy are some of the other concerns that need to be taken care of while attending a patient with seizure. The latter causes belong to the so-called group of "acute symptomatic seizure". Thirdly, known epileptic patients with COVID-19 may experience increased frequency and severity of seizures, particularly because of threshold lowering that remains associated with fever. A patient (mentioned above) with the encephalopathic presentation of COVID-19 had underlying gliosis in the right temporal region resulting from an old embolic stroke. He received prophylactic antiepileptic given the probability of subclinical seizures in the background of a structural brain lesion [17]. Seizures supposedly would complicate the clinical situation by the agency of skeletal muscle injury, which is already a well-known manifestation of the disease. The fact that neurological complications are more frequent in severely ill patients and also cardiovascular risk factors are predictors of severity, the drug interaction potential of several antiepileptic drugs would deserve attention in such clinical situations. Therefore from a neurologist's perspective, seizure in a COVID-19 patient will have some important implications both from diagnostic as well as therapeutic perspectives.

Symptoms of skeletal muscle damage often associated with liver and kidney involvement have already been noted. The reported incidence is 10.7%, and like other neurological features, this one is also associated with a severe form of the illness [10]. It can be speculated that patients with pre-existing renal or hepatic impairment will be highly vulnerable to develop features of multi-organ failure in the backdrop of skeletal muscle injury. Muscle enzymes, including creatine kinase and lactate dehydrogenase (LDH), are seen to be highly elevated in the symptomatic patients - an observation that confirms muscle membrane damage. The exact mechanism of muscle damage, however, has not been established. Possibilities include viral muscle invasion through ACE-2 receptor tropism and immune-mediated muscle fiber damage. Further studies will be required to elucidate the mechanisms underlying skeletal muscle injury in COVID-19.

It has been reported that hypogeusia, as well as hyposmia, are fairly consistent symptoms of SARS-CoV-2 infection [10, 19]. Hypoplasia has also been reported but infrequently. These are all categorized as manifestations of peripheral nerve involvement, while another reported feature is neuralgia. However, noteworthy is that the olfactory nerve is considered part of the CNS, and hyposmia may actually be a reflection of olfactory bulb involvement rather than peripheral neuropathy. Similarly, hypoplasia, if due to optic neuropathy, may reflect CNS manifestation because optic nerve, as per classical teaching, is an extension of the brain.

Post-infective neurological complications

With more number of patients recovering from the SARS-CoV-2 infection, it is imperative that post-infective complications would draw attention with time. CNS demyelinations have been documented previously following coronavirus infection [20]. An early report of Guillain-Barre syndrome (GBS) is available from China, although there is a concern regarding the causality in this particular case [21]. The patient developed typical symptoms of SARS-CoV-2 infection after seven days of hospitalization for GBS. Retrospective analysis, however, supports that she might have been harboring the infection since the beginning, as reflected in her blood counts (lymphocytopenia and thrombocytopenia). The authors duly speculate that the initial symptoms may have been too mild to be detected in this patient (as fever is present in less than half of the cases during the initial phase). A very recent correspondence describes five cases of GBS collected from three hospitals of northern Italy among 100 to 1200 cases of SARS-CoV-2 infection over three weeks [21]. Three of these cases fit criteria for the axonal variant of GBS, while the remaining two had prolonged distal latencies suggesting demyelinating neuropathy. Although each of these cases had a usual latency of five to ten days before the onset of neurological symptoms, one of the patients, similar to the previous one reported from China, was found negative on viral reverse transcription-polymerase chain reaction (RT-PCR) at the outset only to be detected positive subsequently. This observation is ominous because of two reasons: (1) there is a chance of inadvertent exposure to the infectious virus in the neurology ward both for attending health care staffs and other patients; (2) GBS is a disorder known to rapidly affect respiratory muscles particularly if bulbar involvement sets in which can cause sudden poorly explained worsening of a patient's status if the diagnosis of COVID-19 has not already been established.

Another report from China describes a case of acute myelitis, possibly affecting the cervical spinal cord, as evidenced by the clinical features, in a known patient of SARS-CoV-2 infection [22]. In this particular description also the neurological symptoms were co-incident with the febrile period of the illness pointing towards para-infectious demyelination rather than post-infective complication in true sense. Lymphocytopenia accompanied by raised markers of inflammation (CRP and procalcitonin) was documented by the treating physicians. The patient received anti-viral therapy along with immune-suppressive and recovered from his limb weakness. Although the literature is scarce at this point in time, the idea that SARS-CoV-2 can cause para-/post-infective complications affecting the neuroaxis at different levels seems realistic, and supposedly patients with the inflammatory storm will be more likely to manifest this. Additionally, since about 10% of hospitalized patients need assistance in intensive care wards, neurological monitoring must also be aimed at verifying the onset of the so-called "critical illness neuro-myopathy" type PNS problems. These issues are known to delay weaning from ventilation and pose a significant burden on the health care delivery system.

Patients with neurological co-morbidity

During this hour of the pandemic, another group of patients will deserve the attention of neurologists. This group comprises of neurologically ill patients who have already been in follow-up for some time. The need for different groups of patients will vary according to the nature of their illness. Data is insufficient at this point in time to conclude if chronic neurology patients are more predisposed to acquire infection. However, patients with reduced mobility and those on immune-suppression therapy may be anticipated to be more susceptible to infection.

Recent correspondence has already discussed the challenges that will be faced by dementia patients during this period of social distancing and home isolation [23]. Obviously the crisis will be more for those subjects who depend on others for daily life activities. The situation will be similar for patients with prominent motor problems. Due to their restricted mobility, social distancing is supposed to affect them for worse. Reduced mobility and dementia may also predispose a patient to have a viral infection. So once again, the association becomes two-way.

Contemporary neurology has seen wide use of immune-suppressive agents to combat several disorders of both CNS and PNS. The prototypical among these disorders is multiple sclerosis that calls for long term immunosuppression. The data so far on COVID-19 reveals that old age, as well as immune-dysregulated subjects, are more likely not only to acquire the infection but also to manifest increased severity. Therefore, similar to the oncology ward, patients in the neurology ward admitted receiving cyclic immunosuppressive drugs need attention. Appropriate caution has to be practiced while dealing with such cases both from the part of the neurologist as well as the patient's relatives. A recent article on this topic recommends that the benefits of continuing immunotherapy in patients with multiple sclerosis (MS) and related disorders may outweigh the risks of medication withdrawal in the apprehension of COVID-19. This is particularly because most infections, as in the general population, are anticipated to be mild an2d self-limiting. However, the authors emphasize the need for individualized decision-making in such circumstances because one size" may not fit all, and some of the patients may land up in severe infection leading to discontinuation of therapy [24]. 

Long term impact

With the growing number in recovery from SARS-CoV-2 infection, rehabilitation issue is supposed to become a crucial one. Personal communication with neurologists working in the field in Italy reveals that there is early evidence for the need for rehabilitation, including neurological aspects, in clinically recovered patients. It can be assumed that the psycho-social effects of long term social distancing and home isolation will require adequate psychological rehabilitation measures as the pandemic will start waning off.

Since the onset of the SARS-CoV-2 outbreak, if there is one pulmonary manifestation that has received maximum focus, it is ARDS. Evidence suggests a significant percentage of ARDS survivors may suffer long-term cognitive impairment [25]. Several factors, including mechanical ventilation, have been observed to cause a decline in higher brain functions following ARDS. Acute injury to the blood-brain barrier has been implicated as the underlying mechanism for cognitive impairment following ARDS. The effect of such injury may be amplified if there is a pre-existing cognitive impairment that corresponds to chronic blood-brain barrier damage. Patients with brain injury, on the other hand, have been found to develop neurogenic pulmonary edema. Therefore, it is postulated that the so-called brain-lung axis works both ways. The above observations are particularly relevant in the present circumstances, given the need for mechanical ventilation in the majority of the severely affected COVID-19 patients. As the pandemic continues to unfold, the number of people getting off mechanical ventilation will rise, and long-term cognitive outcomes will come into view. It can be anticipated that not only we shall witness cognitive decline lasting for months in this group of patients, but also some of them may progress to premature onset of dementia.

Over time, the need to raise awareness among the neurologists is being increasingly recognized. The case record protocol for "First Few Cases (FFX) and their close contacts" published by WHO has a separate column to mention the neurological findings in addition to respiratory signs and symptoms [26]. This underlines the importance of recognizing the neurological manifestation of the illness. A recently published guideline from China lists some important precautionary measures that need to adapt by the neurologists to combat the pandemic [27]. The bottom line is to have a high index of clinical suspicion, particularly when working in an endemic zone. This will not only help early initiation of therapy but also will halt the chain of transmission. 

## Conclusions

The pandemic of COVID-19 presents for a neurologist some unique challenges. We observe that SARS-CoV-2 may have various neurological manifestations, and in many cases, neurological features may precede typical respiratory symptoms. Holistic knowledge of the spectrum of neurological consequences of COVID-19 is important to get a hold on the spread of the virus. The most vital clinical information which we gather is that impaired consciousness may be a presenting feature of COVID-19, and therefore, a high index of suspicion for such patients will be the key to prevent or, at least, lessen exposure to health care providers and other patients. With the gradual subsidence of the outbreak, it can be anticipated that several post-infectious complications will surface up while rehabilitation measures will also deserve attention. Adequate caution has to be practiced while managing chronic neurology patients, particularly those requiring immune-modulator therapy since formulated guidelines are lacking at this point. The above summary of the neurological manifestations of COVID-19 will help neurologists have a basic preparation, which is of utmost importance to prevent horizontal infections.
